# Musculoskeletal symptoms and biopsychosocial factors among nursing students: implications for protecting the future health workforce

**DOI:** 10.3389/fpubh.2026.1982971

**Published:** 2026-10-07

**Authors:** Kübra Okuyucu, İbrahim Topuz, Gözde Yıldız Daş Geçim

**Affiliations:** 1 Department of Physiotherapy and Rehabilitation, Amasya University, Amasya, Türkiye; 2 Department of Public Health Nursing, Kütahya Health Sciences University, Kütahya, Türkiye; 3 Department of Public Health Nursing, Amasya University, Amasya, Türkiye

**Keywords:** biopsychosocial factors, musculoskeletal disorders, nursing students, occupational health, prevention, public health

## Abstract

**Background:**

Musculoskeletal disorders are an important occupational health concern among healthcare professionals and may emerge before entry into the workforce. Identifying musculoskeletal symptoms and their biopsychosocial correlates among nursing students may provide opportunities for early prevention and health promotion.

**Objective:**

This study aimed to investigate the prevalence of musculoskeletal symptoms and identify associated biopsychosocial factors among nursing students in Türkiye.

**Methods:**

A cross-sectional study was conducted among 213 nursing students (77% female) in Türkiye. Data were collected through an online survey between February and April 2022. Musculoskeletal symptoms, physical activity, perceived stress, biopsychosocial wellbeing, and coping behaviors were assessed using validated instruments. Associations were examined using chi-square tests and independent-samples *t*-tests; logistic regression analyses, using a simplified set of six predictors (age, BMI, gender, physical activity, total perceived stress, and total biopsychosocial response score) and Firth's penalized-likelihood method, were performed to identify factors associated with musculoskeletal symptoms.

**Results:**

Overall, 62% of nursing students reported musculoskeletal symptoms during the previous 12 months. The most frequently reported symptoms were in the upper back (41%), followed by the low back and neck (33% each). High physical activity showed a significant association with lower odds of upper back symptoms (OR = 0.19, *p* = 0.047) and a borderline association with lower odds of any 12-month musculoskeletal symptom (OR = 0.25, *p* = 0.060). Female students had higher odds of neck, shoulder, and upper back symptoms than male students (OR = 0.05–0.19 for male vs. female). No stress or biopsychosocial measure was significantly associated with any 12-month musculoskeletal outcome; findings for the hip/thigh region should be considered exploratory given a low events-per-variable ratio.

**Conclusions:**

Musculoskeletal symptoms were highly prevalent among nursing students, highlighting the importance of addressing musculoskeletal health before entry into the nursing workforce. The findings suggest that physical factors may be more relevant to symptom occurrence than stress or biopsychosocial wellbeing in this sample. Although a biopsychosocial framework provides a useful perspective for understanding musculoskeletal health, further longitudinal research is needed to clarify the role of stress and wellbeing and to inform targeted interventions.

## 1 Introduction

Musculoskeletal disorders (MSDs) are a major public health concern associated with pain, activity limitation, reduced quality of life, and disability. Although traditionally considered an occupational and age-related problem, current evidence indicates that musculoskeletal symptoms may also affect young adults and can have consequences for education and future work participation (1–5). Given their complex and multifactorial etiology, MSDs should be addressed within a biopsychosocial framework that integrates biological, psychological, and social determinants (6–8).

Nursing students may be particularly vulnerable because clinical education introduces exposure to prolonged standing, patient handling (9), repetitive movements, awkward postures, and other physical demands (5, 10), alongside academic and clinical stressors (11, 12). Evidence from national and international studies have reported a substantial prevalence of musculoskeletal complaints among nursing students, with prevalence estimates reaching up to 83% (9, 10, 13, 14), and identified physical demands, lifting and bending, physical activity, and clinical placement characteristics as relevant factors (9, 10).

MSDs are influenced by a combination of individual, occupational, and psychosocial factors (14–16). The biopsychosocial model proposed by Engel (8) emphasizes the interplay of biological (e.g., posture, workload), psychological (e.g., stress, coping strategies), and social (e.g., environmental conditions, peer support) factors. However, existing studies have often examined physical, psychological, or psychosocial factors separately, limiting understanding of how these factors coexist in nursing students. A comprehensive assessment may help clarify the multifactorial nature of musculoskeletal symptoms among nursing students and identify opportunities for early prevention.

Accordingly, this study aimed to investigate the prevalence of self-reported musculoskeletal symptoms among nursing students in Türkiye and to identify associated demographic, physical, psychological, and biopsychosocial factors. By examining these factors before entry into the nursing workforce, the study seeks to provide evidence relevant to early occupational health prevention and the development of health workforce strategies that promote musculoskeletal health and wellbeing among future nurses. The findings may inform nursing education and institutional policies aimed at identifying modifiable risk factors and preparing future nurses for safer and healthier working lives.

## 2 Methods

### 2.1 Study design and population

This cross-sectional study was conducted between February and April 2022 using a self-administered online survey. The target population comprised undergraduate nursing students enrolled at universities in Türkiye. Participants were recruited from universities located in nine cities across five geographical regions of Türkiye. Of the 213 participants, 114 (53.5%) were from Amasya (Black Sea region), 47 (22.1%) from Kütahya (Aegean region), 34 (16.0%) from Kocaeli, Istanbul, Balikesir, and Kirklareli (Marmara region), 14 (6.6%) from Adiyaman and Sirnak (Southeastern Anatolia), and 4 (1.9%) from Antalya (Mediterranean region). Thus, participants were recruited across five geographical regions, although the distribution of participants was uneven, with the largest proportion recruited from the Black Sea region. A non-probability convenience and snowball sampling strategy was used. Sample size was calculated based on the number of predictors in the logistic regression model using an anticipated medium effect size of 0.15, α = 0.05, and power = 0.80. For seven predictors, the minimum required sample was 103 participants; the final sample of 213 exceeded this requirement (17). The *a priori* sample-size calculation was based on the originally planned model including seven predictors and indicated a minimum sample size of 103 participants; during the subsequent analysis, the predictor set was reduced to six variables to obtain more parsimonious models and minimize the risk of overfitting.

Nursing education in Türkiye progresses from predominantly classroom- and laboratory-based learning in the first year to increasing clinical exposure in subsequent years. During the second and third years, students spend increasing time in hospital wards providing basic patient care and developing technical skills. Senior students undertake extended clinical placements involving more complex patient care, prolonged standing and walking, frequent manual handling, and greater decision-making responsibilities (18, 19).

### 2.2 Selection criteria

Participants were eligible if they were ≥18 years, enrolled in an undergraduate nursing program at a university in Türkiye, voluntarily consented to participate, and had internet access. Students who could not provide informed consent or could not complete the online survey because of severe cognitive impairment or insufficient proficiency in Turkish were excluded. Pre-existing musculoskeletal disorders were not an exclusion criterion; students with and without previous symptoms were eligible. Students from multiple universities across Türkiye were eligible to enhance sample diversity.

### 2.3 Data collection

The survey assessed musculoskeletal symptoms and individual and nursing education-related risk factors using a participant information form and five validated instruments: the extended nordic musculoskeletal questionnaire (NMQ-E) (20, 21), perceived stress scale (PSS), bio-psycho-social response scale (BPSRS) and coping behavior inventory (CBI) (22), international physical activity questionnaire (IPAQ) short form (23, 24).

Before data collection, the online survey was pretested by nine nursing students and three academic staff members with expertise in nursing and survey methodology. Pilot participants were not included in the final sample, and minor revisions were made to improve clarity and contextual appropriateness.

Following ethical approval, invitations were distributed through academic staff, institutional networks, and national student and academic networks to undergraduate nursing students at universities across Türkiye. The anonymous questionnaire was administered through Google Forms. Participants first reviewed the information sheet and electronic consent form and provided electronic informed consent before accessing the questionnaire. No personally identifiable information was collected. Data were stored on a secure, password-protected platform accessible only to the researchers. Participation was voluntary, confidential, and without incentives. Periodic reminders were sent to academic staff to encourage participation and improve response rates. Distribution across multiple universities and geographical regions aimed to enhance sample diversity.

### 2.4 Data collection tools

#### 2.4.1 Participant information form

The researcher-developed form collected age, gender, weight, height, and year of study (1st, 2nd, 3rd, or 4th year). BMI was calculated from weight and height and entered into analyses as a continuous variable (9, 13).

#### 2.4.2 International physical activity questionnaire (IPAQ) short form (7 items)

The Turkish version assessed physical activity using seven questions concerning activity type, duration, and frequency. Participants were categorized as having low, moderate, or high physical activity levels (23, 24).

#### 2.4.3 Extended nordic musculoskeletal questionnaire (NMQ-E)

The Turkish version assessed pain or discomfort in nine body regions (neck, shoulders, upper back, elbows, wrists/hands, lower back, hips/thighs, knees, and ankles/feet) across lifetime, 12-month, and 1-month periods (20, 21). Participants also reported whether symptoms affected daily activities, resulted in absence from school, and were perceived as related to nursing education/care activities, leisure activities, or other causes such as falls or accidents.

#### 2.4.4 Perceived stress scale (PSS)

The Turkish-adapted PSS consists of 29 items across six domains: patient care (8 items), teachers/nursing personnel (6), assignments/workload (5), peers/daily life (4), professional knowledge/skills (3), and clinical environment (3). Items are rated from 0 to 4, yielding a total score of 0–116, with higher scores indicating greater stress (22, 25).

#### 2.4.5 Bio-psycho-social response scale (BPSRS)

The Turkish version comprises 21 items rated from 0 to 4, with total scores ranging from 0 to 84. Higher scores indicate more symptoms and poorer biopsychosocial status. It includes physical (eight items), emotional (seven items), and social-behavioral (six items) domains (22).

#### 2.4.6 Coping behavior inventory (CBI)

The Turkish version consists of 19 items rated from 0 to 4 across avoidance (6 items), problem-solving (6), optimistic (4), and transference (3) coping behaviors. Higher scores indicate more frequent use of the corresponding coping behavior.

In the present sample, Cronbach's α was 0.98 for PSS, 0.97 for BPSRS, and 0.92 for CBI (22). The internal consistency observed in this sample (α = 0.98 for PSS, 0.97 for BPSRS) exceeded that reported in the original Turkish validation study (22), which may reflect item redundancy within these scales or an acquiescence response pattern in this sample, and should be considered when interpreting the strength of associations involving these measures.

### 2.5 Data analysis

Data were analyzed using SPSS version 24.0 (IBM Inc., Armonk, NY, USA). Descriptive statistics were used to summarize participant characteristics and the prevalence, severity, and impact of musculoskeletal symptoms. The distribution of continuous variables was assessed using histograms, Q–Q plots, and the Shapiro–Wilk test. Continuous variables were considered approximately normally distributed based on visual inspection and the results of the normality assessments.

Independent-samples *t*-tests were used to compare normally distributed continuous variables between participants with and without musculoskeletal symptoms. Categorical variables, including gender and physical activity level, were compared using chi–square tests. Spearman's rank correlation was used to assess associations between continuous biopsychosocial variables and musculoskeletal symptom prevalence. Logistic regression models examined associations between musculoskeletal symptoms and multiple predictors, with odds ratios (ORs) and 95% confidence intervals (CIs) reported. The variables were selected based on their theoretical relevance to musculoskeletal symptoms and their statistical significance in univariate analyses (*p* < 0.20) and were entered into the multivariable models using the forced-entry method. Linearity of continuous predictors in the logit was assessed using the Box–Tidwell procedure, and all continuous predictors met this assumption. Year of study was excluded from regression models because of its strong correlation with age and lack of association with musculoskeletal symptoms. Because several outcomes had a low number of events relative to the number of predictors, all six logistic regression models were fitted using Firth's penalized-likelihood logistic regression to reduce small-sample bias, and predicted probabilities are reported alongside odds ratios where estimates were extreme. Statistical significance was set at *p* < 0.05 (two-tailed) for all analyses. Listwise (complete-case) exclusion was used for the logistic regression models. All participants completed the NMQ-E; therefore, no participants were excluded because of missing musculoskeletal symptom data in the descriptive and bivariate analyses. Physical activity had the largest amount of missing data (*N* = 111 complete of 213); because physical activity was included as a predictor in every regression model, all six logistic regression models, including the model for any musculoskeletal symptom, were fitted on this same *N* = 111 complete-case subsample. Missing data were also observed for the total PSS (75 missing; *N* = 138 complete) and the total BPSRS (88 missing; *N* = 125 complete). However, all participants with missing PSS or BPSRS data also had missing physical activity data. Thus, physical activity was the main limiting factor for the analytical sample, and all six logistic regression models, including the model for any musculoskeletal symptom, were fitted using the same complete-case sample of *N* = 111 participants with complete data for all model predictors. No adjustment for multiple comparisons was applied, and the findings were interpreted cautiously considering the increased risk of Type I error associated with multiple statistical comparisons.

### 2.6 Ethical aspects

The study was approved by the Non-Interventional Clinical Research Ethics Committee of Amasya University (4 February 2022; Approval No. E-30640013-050.01.04-55787). Before completing the anonymous questionnaire, all participants received an information sheet describing the purpose and procedures of the study, the voluntary nature of participation, confidentiality and anonymity of the collected data, the right to withdraw from the study at any time without penalty, and the approximate time required to complete the survey. Electronic informed consent was obtained from all participants before participation. The study was conducted in accordance with the Declaration of Helsinki. Artificial intelligence was not used in any part of the study.

## 3 Results

A total of 213 students completed the survey. As the number of invited students was unknown, a response rate could not be calculated. Participant characteristics are presented in Table 1. Mean age was 21.05 years (SD: 1.77; range: 18–35), and 77% were women. Among students who completed the physical activity questionnaire, most reported low or moderate physical activity levels.

**Table 1:** Characteristics of the participants (*N* = 213).

Variables	*N* (%) or mean (SD)	Range
Year of study		
1	18 (9%)	-
2	88 (41%)	-
3	30 (14%)	-
4	77 (36%)	-
Age (years)	21.05 (1.77)	18–35
Height (cm)	166.24 (8.81)	150–195
Weight (kg)	61.41 (12.8)	43–113
BMI (kg/m^2^)	22.08 (3.31)	15.89–38.22
Gender		
Male	48 (23%)	-
Female	165 (77%)	-
Physical activity level (*n* = 111)		
Low	50 (45%)	-
Medium	48 (43%)	-
High	13 (12%)	-

### 3.1 Prevalence and severity of musculoskeletal symptoms

Lifetime, 12-month, and 1-month prevalence was 80% (*n* = 171), 62% (*n* = 132), and 44% (*n* = 93), respectively. As shown in Table 2, 12-month prevalence were most common in the upper back (41%), followed by the low back and neck (33% each) and shoulders (29%). Symptoms affected daily activities in 32% of students, while only seven students (3%) missed clinical practice or lectures due to musculoskeletal symptoms.

**Table 2:** 12-month prevalence of musculoskeletal symptoms, severity and impact (*n* = 213).

Body area / measure	Musculoskeletal symptom prevalence in the last 12 months (*n* = 213) *N* (%)	Severity (affected daily life routines during the last 12 months) (*n* = 213) *N* (%)	Sick leave (away from school during the last 12 months) (*n* = 213) *N* (%)
Total number of students	**132 (%62)**	**69 (32%)**	7 (3%)
Body area			
Neck	**70 (33%)**	16 (8%)	0
Shoulders	**61 (29%)**	19 (9%)	2 (1%)
Upper back	**88 (41%)**	25 (12%)	3 (1%)
Elbows	9 (4%)	0	0
Wrists/hands	25 (12%)	3 (1%)	0
Low back	**71 (33%)**	33 (16%)	5 (2%)
Hips/thighs	24 (11%)	7 (3%)	0
Knees	44 (21%)	15 (7%)	1 (1%)
Ankles/feet	29 (14%)	15 (7%)	2 (1%)

Percentages may exceed 100% as participants were allowed to select more than one response. Bold values indicate musculoskeletal symptom prevalence rates >29% in the last 12 months.

Leisure activities were the most commonly perceived cause of upper back (47%), neck (41%), low back (39%), and shoulder (40%) symptoms, while only four students (4.5% of those with upper back symptoms) attributed their symptoms to clinical practice.

### 3.2 Perceived stress, biopsychosocial responses, and coping behaviors

Participants' stress, biopsychosocial responses, and coping behaviors were evaluated using the PSS, BPSRS, and CBI (Table 3). The mean total PSS score was 55.09 (SD = 34.83), with highest stress reported from patient care and interactions with teachers/nursing personnel. The mean total BPSRS score was 29.48 (SD = 22.10), predominantly reflecting emotional and social responses. Among coping behaviors, problem-solving strategies were most frequently used (mean = 15.03, SD = 6.45), followed by optimistic, avoidance, and transference behaviors.

**Table 3:** Scores of perceived stress (PSS), biopsychosocial responses (BPSRS), and coping behaviors (CBI).

PSS	Mean	Std. deviation	Range
Total Stress	55.09	34.830	0–116
Stress from lack of professional knowledge and skills	6.07	3.685	0–12
Stress from taking care of patients	16.16	9.793	0–32
Stress from assignments and workload	10.32	6.324	0–20
Stress from teachers and nursing personnel	10.91	7.422	0–24
Stress from the clinical environment	5.96	3.823	0–12
Stress from peers and daily life	6.06	5.227	0–16
BPSRS			
Total BPS	29.48	22.096	0–84
Social BPS	10.09	6.812	0–24
Emotional BPS	11.55	8.173	0–28
Physical BPS	7.91	8.845	0–32
CBI			
Optimistic coping behaviors	8.98	3.189	0–16
Transference behaviors	6.43	2.902	0–12
Problem solving behaviors	15.03	6.448	0–24
Avoidance behaviors	7.30	6.470	0–24

The number of valid observations varied across PSS and BPS subscales because of missing data. Total PSS and BPS scores were calculated using complete cases; therefore, subscale means may not sum exactly to the corresponding total score mean.

### 3.3 Risk factors for musculoskeletal symptoms

Factors were compared between musculoskeletal symptoms ‘Yes' and ‘No' groups. Lower BMI was associated with 12-month neck (*p* = 0.02), low back (*p* = 0.01), and wrist/hand (*p* = 0.03) symptoms. Female students reported more neck (*p* = 0.01), shoulder (*p* < 0.001), and upper back (*p* = 0.002) symptoms. Low physical activity was associated with upper back symptoms (*p* = 0.01). Knee symptoms showed a borderline association with total stress (*p* = 0.05), lack of professional knowledge and skills (*p* = 0.004), assignments and workload (*p* = 0.04), and teachers/nursing personnel (*p* = 0.01), with lower stress scores among students reporting symptoms. No significant differences were found for the remaining factors.

All factors except year of study were entered into the logistic regression models (Table 4). Due to strong correlation with age and lack of association with musculoskeletal symptoms, year of study was excluded. Multicollinearity was not a concern (all VIF <5; tolerance >0.2). Female gender and low physical activity were used as reference categories. Because several outcomes had few events relative to the number of candidate predictors, the regression models were simplified to six core predictors (age, BMI, gender, physical activity, total stress, and total biopsychosocial response score) and fitted using Firth's penalized-likelihood logistic regression. High physical activity showed a borderline inverse association with any 12-month MSD (OR=0.25, 95% CI: 0.06–1.06, *p* = 0.060). Male students had markedly lower odds of neck (OR=0.05, 95% CI: 0.01–0.39, *p* = 0.004), shoulder (OR=0.05, 95% CI: 0.01–0.34, *p* = 0.002), and upper back (OR=0.19, 95% CI: 0.05–0.70, *p* = 0.013) symptoms than female students, consistent with the bivariate findings above; predicted probabilities at mean covariate values were 44.7% (female) vs. 3.9% (male) for neck, 45.4% vs. 4.0% for shoulder, and 56.1% vs. 19.2% for upper back symptoms. Older age was independently associated with higher odds of neck symptoms (OR=1.46, 95% CI: 1.04–2.06, *p* = 0.029). High physical activity was also associated with lower odds of upper back symptoms (OR=0.19, 95% CI: 0.04–0.98, *p* = 0.047). No predictor reached significance for low back symptoms, although lower BMI showed a borderline association (OR=0.87, 95% CI: 0.75–1.01, *p* = 0.061). In the hip/thigh model, no predictor reached significance (all *p* > 0.05); given this model's low events-per-variable ratio (2.0), these results should be considered exploratory.

**Table 4:** Firth penalized-likelihood logistic regression results for predictors of 12-month musculoskeletal symptoms from the final (simplified) models (*N* = 111); significant (*p* < 0.05) and one borderline (*p* < 0.10) associations are shown.

Outcome (events, EPV)	Predictor	OR (95% CI)	*p*
Any musculoskeletal symptoms (79, EPV 11.3)	PA: high vs. low	0.25 (0.06–1.06)	0.060^‡^
Neck (39, EPV 5.6)	Age	1.46 (1.04–2.06)	0.029^*^
	Male vs. Female	0.05 (0.01–0.39)	0.004^**^
Shoulder (39, EPV 5.6)	Male vs. Female	0.05 (0.01–0.34)	0.002^**^
Upper back (54, EPV 7.7)	Male vs. Female	0.19 (0.05–0.70)	0.013^*^
	PA: high vs. low	0.19 (0.04–0.98)	0.047^*^
Low back (40, EPV 5.7)	(none significant at *p* < 0.05)	—	—
Hips/thighs—exploratory (14, EPV 2.0)	(none significant at *p* < 0.05)	—	—

Predictors: age, BMI, gender (male vs. female, ref.), physical activity (two dummy-coded terms: moderate vs. low [ref.], and high vs. low [ref.]; only high-vs.-low reached significance for any outcome and is shown), total PSS stress score, total BPSRS score. *N* = 111 for all six models.

OR, odds ratio; CI, confidence interval.

^*^Significance level, *p* value <0.05.

^**^Significance level, *p* value <0.01.

^‡^Borderline association, *p* value <0.10 (not significant at the *p* < 0.05 threshold).

A conceptual model was developed to illustrate the links between biopsychosocial factors, musculoskeletal symptoms and educational strategies in nursing students (Figure 1). Relationships are theoretically derived and were not empirically tested for directionality or causality in this cross-sectional study.

**Figure 1:**
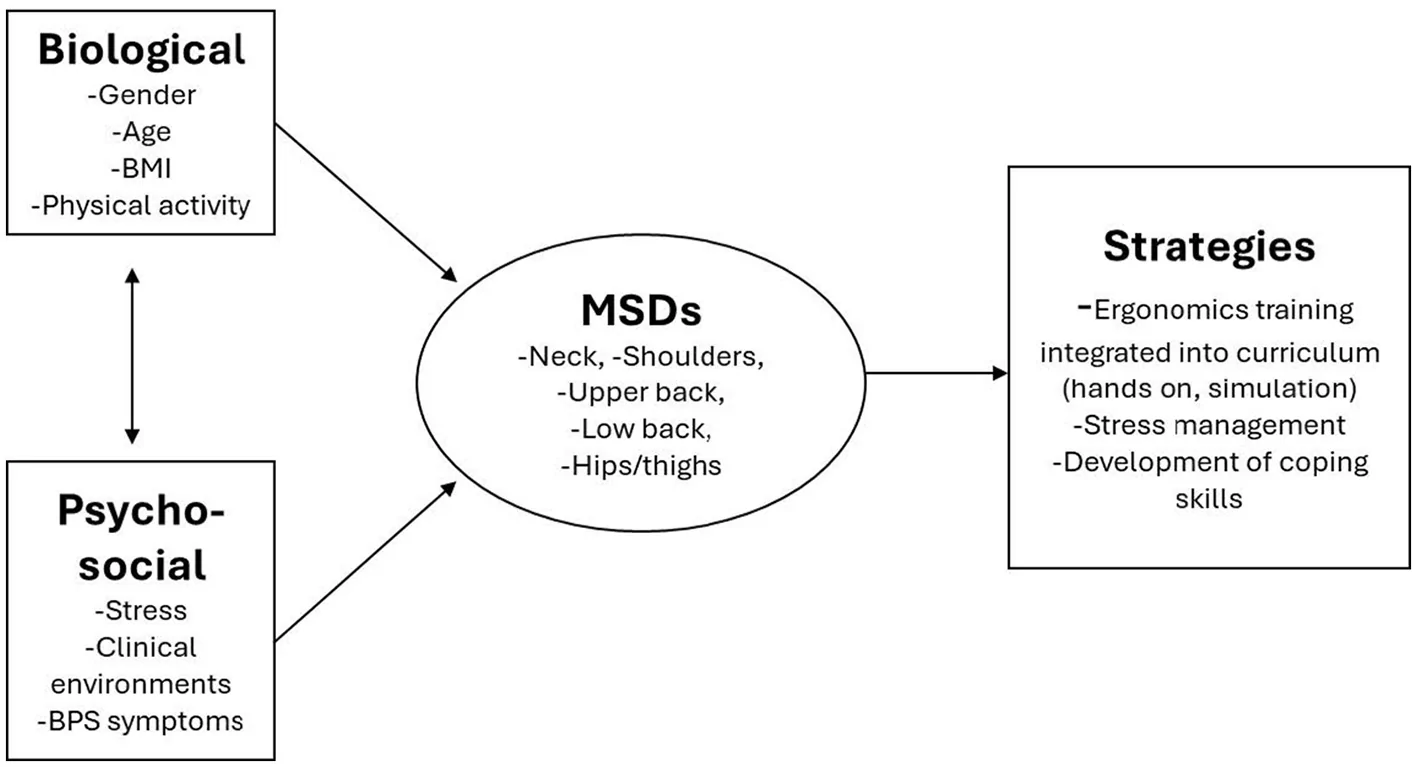
Conceptual framework illustrating hypothesized relationships between biopsychosocial factors, MSDs, and educational strategies in nursing students. Relationships are theoretically derived and were not empirically tested for directionality or causality in this cross-sectional study.

## 4 Discussion

The present study found that 62% of nursing students reported musculoskeletal symptoms in at least one body region during the preceding 12 months, with the upper back, low back, and neck being the most frequently affected regions. Importantly, nearly one-third of students reported that their symptoms affected daily activities. These findings indicate that musculoskeletal symptoms are already common during undergraduate nursing education rather than emerging only after entry into professional nursing practice.

The prevalence observed in the present study is broadly consistent with more recent evidence from nursing-student populations. A study involving 711 Dutch nursing students reported musculoskeletal complaints in 79% of participants and identified physical job demands, lifting and bending, and need for recovery as associated factors (9). In another recent study, more than half of undergraduate nursing students reported musculoskeletal pain during the previous year, with inadequate physical activity and strenuous clinical placements associated with symptoms (10). Although prevalence estimates vary according to measurement instruments, anatomical definitions, educational systems, and recall periods, the consistency of these findings supports the interpretation that musculoskeletal symptoms represent a relevant health issue during nursing education. For example, Türkiye offers a four-year program with longer clinical placements and more intensive patient-handling training, in other countries nursing education is typically three years (e.g., England, Germany, Poland, Azerbaijan). This may increase exposure to prolonged standing, manual handling, and psychosocial stress, highlighting the need for context-specific preventive strategies to support students' health and future workforce resilience.

The distribution of symptoms observed in the present study, particularly the prominence of the upper back, low back, and neck, is also broadly consistent with findings from nursing-student populations, with the upper back being the most frequently affected body region in the present study, with a prevalence of 41%. Similar patterns have been reported among nursing students, although the most affected anatomical regions have varied across studies. Differences in clinical exposure, educational settings, task allocation, physical activity levels, and measurement methods may partly explain these variations (9, 14). The predominance of upper back symptoms may reflect prolonged static postures during documentation and screen-based tasks, repetitive bag or equipment carrying, and the seated/forward-flexed postures common during classroom and laboratory instruction, in addition to clinical exposures such as assisting with patient mobility, repositioning patients, and performing procedures at bedside. Differences in students' physical activity levels and the types or frequency of clinical tasks they perform may also contribute to variation in symptom prevalence across body regions. Findings from practicing nurses provide a broader occupational context for interpreting these results. Evidence from practicing nurses similarly identifies the low back, neck, and shoulders among the body regions most frequently affected by work-related musculoskeletal disorders (5). Although nursing students cannot be considered equivalent to practicing nurses, the overlap in affected anatomical regions suggests that some musculoskeletal problems commonly observed in nursing practice may already be present during professional preparation. However, comparisons with practicing nurses should be interpreted cautiously because students have different levels of clinical exposure, work responsibilities, and cumulative occupational loading. Therefore, comparisons with nursing-student populations provide the more appropriate basis for interpreting the present findings, whereas evidence from practicing nurses may help contextualize the potential relevance of these symptoms to future professional practice.

Importantly, the findings also indicate a distinction between symptom prevalence and functional impact across body regions. Although upper back symptoms were the most prevalent (41%), low back symptoms were more likely to affect daily activities (16% vs. 12%) and result in absence from clinical practice or lectures (2% vs. 1%). This distinction suggests that prevalence alone may not identify the regions with the greatest functional or occupational impact, and that preventive strategies should consider severity and impact, not frequency alone, when prioritizing musculoskeletal health interventions for nursing students.

Interestingly, students more frequently perceived their musculoskeletal symptoms as being related to leisure activities rather than clinical practice. This finding suggests that musculoskeletal symptoms among nursing students may reflect exposures occurring across multiple aspects of daily life rather than clinical training alone. However, these perceptions are self-reported and should not be interpreted as evidence that clinical practice does not contribute to musculoskeletal symptoms.

In this study, high physical activity showed a borderline inverse association with overall symptom prevalence in the simplified regression model (OR = 0.25, 95% CI: 0.06–1.06, *p* = 0.060), and was significantly associated with lower odds of upper back symptoms specifically (OR = 0.19, *p* = 0.047), consistent with evidence showing reduced musculoskeletal symptoms with regular exercise (26). Similar findings have been reported in student nurses, where higher exercise frequency was linked to lower musculoskeletal complaints and stress (27). However, some studies found no association between physical activity and pain (28), possibly due to differences in measurement methods, intensity, and duration of activity. However, the association observed in the present study should not be interpreted as evidence that greater physical activity directly prevents musculoskeletal symptoms, particularly because of the cross-sectional design and the relatively small number of participants with complete physical activity data (*N* = 111).

The current study found a positive association between age and neck pain, indicating that older students reported higher levels of symptoms. This is consistent with evidence showing an overall increase in MSD prevalence with age (29), although some studies report greater susceptibility among younger healthcare professionals (28), possibly due to less experience in ergonomic risk management.

In the bivariate analysis the low back symptoms showed a negative association with BMI, though this association was only borderline in the simplified, adjusted regression model (OR = 0.87, 95% CI: 0.75–1.01, *p* = 0.061). This finding contrasts with existing literature, which generally suggests that higher BMI increases mechanical load on the lumbar region. The unexpected result may be explained by behavioral factors, such as students with higher BMI may engage in fewer physically demanding clinical tasks, reducing mechanical exposure. Self-selection may also play a role, as students with pre-existing musculoskeletal complaints may adopt lower-BMI lifestyles independent of pain status. Measurement bias is possible given self-reported height and weight. Reverse causality cannot be excluded, as pain may lead to reduced activity and weight change over time. Given the cross-sectional design, the findings should be interpreted cautiously and requires further investigation in longitudinal samples

Domain-level stress and biopsychosocial subscale scores were not retained as predictors of hip/thigh symptoms in the final regression model; associations observed for individual subscales in preliminary domain-level models did not persist once the predictor set was simplified to reduce overfitting and are not interpreted as genuine findings. However, stress is generally conceptualized in the literature as a potential contributor to MSD (11, 12, 30). This study did not identify a significant association between stress and any MSD outcome. Cross-country differences in clinical workload and organizational conditions may still be relevant to stress-related MSD risk and warrant further investigation with adequately powered samples.

The social domain of the BPSRS includes six items assessing social and behavioral responses to symptoms, such as withdrawal from social or academic activities and reduced peer interaction. Lower scores may reflect social disengagement associated with musculoskeletal symptoms and may indicate a need for combined psychosocial and physical-health support. Taken together, the findings of this study can be interpreted within the biopsychosocial framework, which emphasizes the interaction of biological, psychological, and social factors in the development and experience of musculoskeletal symptoms. From a biological perspective, factors such as physical activity, BMI, age, and exposure to physically demanding clinical tasks may influence musculoskeletal loading and symptom development. From a psychological perspective, stress related to patient care and the clinical environment may influence muscle tension, fatigue, pain perception, and coping responses. From a social perspective, students' social and interpersonal circumstances may affect access to support, coping with academic and clinical demands, and engagement in health-related behaviors. The observed associations between physical activity, BMI, stress, and social biopsychosocial status and regional musculoskeletal symptoms therefore suggest that musculoskeletal symptoms among nursing students are unlikely to be explained by a single factor. Rather, biological, psychological, and social factors may interact with one another and with the clinical learning environment to influence students' musculoskeletal health. This perspective supports the need for multidimensional preventive strategies that combine ergonomic and physical interventions with psychological support and strengthening of social support systems during nursing education. The conceptual model in Figure 1 reflects theoretical relationships drawn from the biopsychosocial literature and this study's cross-sectional associations; it should not be interpreted as an empirically validated causal pathway. Longitudinal studies are needed to test the directionality of the proposed relationships.

### 4.1 Limitation

This study has several limitations that should be considered when interpreting the findings. First, in Türkiye, nursing curricula are standardized by the Higher Education Council which may differ from international programs in terms of structure, curricular emphasis, and clinical placement exposure. As a result, contextual factors such as workload distribution, patient expectations, and clinical environment characteristics may influence students' experiences and should be taken into account when considering the transferability of the findings to other educational or healthcare systems. Second, the gender imbalance in the sample may have affected the observed associations. Additionally, the distribution of participants across years of study was unequal (1st year: 9%; 2nd: 41%; 3rd: 14%; 4th: 36%), which may affect the representativeness of findings for underrepresented cohorts, particularly 1st- and 3rd-year students, and limits generalizability across the full nursing curriculum. Furthermore, although participants were recruited across five geographical regions, the distribution was uneven, limiting the national representativeness and generalizability of the findings. Third, all data were based on self-reported measures, which are subject to recall and response bias. There is also potential for self-selection bias as some students with musculoskeletal symptoms may have been more motivated to participate the study. Additionally, this is a cross-sectional designed study, causal relationships cannot be inferred. Associations observed between factors and musculoskeletal symptom outcomes may reflect underlying patterns rather than directional effects. These findings should therefore be interpreted as associations rather than causal effects, and future longitudinal and experimental studies would allow cause-effect interpretation within the local context. The lack of adjustment for multiple comparisons may have increased the risk of Type I error; therefore, the findings should be interpreted cautiously. Furthermore, because of missing physical activity data, all six logistic regression models were fitted on a complete-case subsample of *N* = 111; even after simplifying the predictor set to six core variables, events-per-variable ratios ranged from 2.0 (hips/thighs) to 11.3 (any MSD), and the hip/thigh model did not converge under standard maximum-likelihood estimation, so its findings should be considered exploratory rather than confirmatory.

### 4.2 Implications

The findings support consideration of musculoskeletal health as an early occupational health priority within nursing education. The high prevalence of self-reported musculoskeletal symptoms among nursing students suggests that preventive approaches may be appropriately initiated during professional preparation, rather than only after entry into the nursing workforce. Nursing curricula could strengthen education on ergonomics, musculoskeletal health, safe patient handling, physical activity, and stress management. Simulation-based training may provide opportunities to reinforce safe postures and handling practices, while interprofessional learning could enhance students' understanding of teamwork, human factors, and psychosocial aspects of occupational safety.

At the institutional and policy levels, early identification and health-promotion strategies could be incorporated into nursing education and broader health workforce initiatives. Although causal relationships cannot be inferred from this cross-sectional study, addressing potentially modifiable physical and psychosocial factors before workforce entry may help establish a culture of occupational safety and contribute to healthier and more sustainable nursing careers. Such preventive approaches may ultimately support efforts to protect health workers and the quality of healthcare services.

## 5 Conclusion

This study found a substantial burden of self-reported musculoskeletal symptoms among nursing students, with physical activity, demographic characteristics, selected stress-related factors, and biopsychosocial wellbeing associated with musculoskeletal outcomes. These findings reinforce the importance of addressing musculoskeletal health during professional education as an early occupational health concern. A biopsychosocial approach that integrates ergonomics, physical activity, stress management, and supportive learning environments may provide a useful foundation for promoting musculoskeletal health and occupational safety before entry into the nursing workforce. From a health workforce perspective, embedding preventive approaches within nursing education may contribute to healthier and more sustainable nursing careers and, ultimately, to the protection of the healthcare workforce. Longitudinal and intervention studies are needed to clarify temporal relationships and determine whether multidimensional preventive programmes introduced during nursing education can reduce musculoskeletal symptom burden and improve wellbeing.

## Data Availability

The data that support the findings of this study are openly available in Mendeley Data at https://data.mendeley.com/datasets/zss7rh4hsv/1, cited as Okuyucu, Kubra (2024), “MSD in nursing students”, Mendeley Data, V1, doi: 10.17632/zss7rh4hsv.1 422 423.
